# A Molecular Dynamics Study of the Solvation Properties of Sugars in Supercritical Carbon Dioxide

**DOI:** 10.3390/molecules30061256

**Published:** 2025-03-11

**Authors:** Alexandrine Lambert, Francesca Ingrosso

**Affiliations:** Laboratoire de Physique et Chimie Théoriques UMR 7019, Université de Lorraine and CNRS, F-54000 Nancy, France

**Keywords:** supercritical solvents, molecular dynamics, solvation, functionalized sugars, cyclodextrins

## Abstract

Among the various strategies used to enhance the solvation power of supercritical carbon dioxide (scCO_2_), the use of CO_2_-philic compounds has been extensively studied over the recent two decades. Given the biocompatibility of this medium, extraction technologies based on scCO_2_ are particularly attractive, and a molecular-level understanding of intermolecular interactions is crucial for optimizing processing conditions. Functionalized sugars and cyclic oligosaccharides, such as cyclodextrins, can be rendered soluble in scCO_2_, opening new avenues for vectorization strategies and supramolecular chemistry in this medium. To support the exploration of CO_2_-philic compounds relevant to these research goals, we conducted a molecular dynamics investigation into the solvation properties of cyclodextrins functionalized with CO_2_-philic groups. We thoroughly analyzed the key solute–solvent interactions and their influence on the cavity shape. Additionally, we provided insights into the solvation behavior of peracetylated α and β-glucose across different regions of the carbon dioxide phase diagram. We were able to confirm the importance of the well-known (acetyl)C–O⋯C(CO_2_) interaction, as the most important signature of CO_2_-philicity of carbonyl compounds. Depending on the substituent, this interaction can be assisted by a cooperative (methyl)_2_HCH⋯O(CO_2_) intermolecular bond. In cyclodextrins, conformational flexibility, with a possible change in the conformation of some pyranose units, was observed in the macromolecular structure. On the other hand, these structural modifications were not present for α- and β-glucose.

## 1. Introduction

The phase diagram of carbon dioxide presents a critical point characterized by a critical temperature of 304.1282 K, a critical pressure of 7.377 MPa and a critical density of 467.6 kg/m^3^ [1,2]. Beyond this threshold, the distinction between liquid and vapor phases disappears, giving rise to a supercritical phase. Supercritical CO_2_ (scCO_2_) has attracted attention as a ’green’ alternative medium for extraction on an industrial scale, given the easier conditions to reach the supercritical phase compared with other fluids, its large availability, non-toxicity, and non-flammability [3,4]. However, the conditions to reach the supercritical phase require high pressure, thus requiring special equipment. In addition, CO_2_ has a low dielectric constant and generally displays poor solubilizing power for polar species [5]. To overcome these limitations, especially with the scope of lowering the working pressure, one possible strategy is to use cosolvents [6].

Another potential approach is to promote the design of compounds containing specific groups known as ’CO_2_-philic’. When incorporated into a molecule or macromolecular structure, these groups facilitate favorable interactions with CO_2_, thereby improving solubility. However, this strategy may raise additional environmental concerns. For cosolvents, conventional organic solvents are typically mixed with CO_2_. One of the first substituents shown to possess an affinity with CO_2_ has been fluorine. However, fluorinated compounds can be expensive and their biological compatibility has been questioned, particularly with respect to the persistence of fluorinated polymers in the environment [7].

The approach of using CO_2_-philic molecules to introduce otherwise insoluble units into the medium led to pioneering work by Beckman’s group, resulting in poly(ether-carbonate) copolymers that remain soluble even at low pressures [4]. In these monomers, the acetate moiety was identified as the unit responsible for CO_2_-philicity [8,9,10].

**Figure 1:**
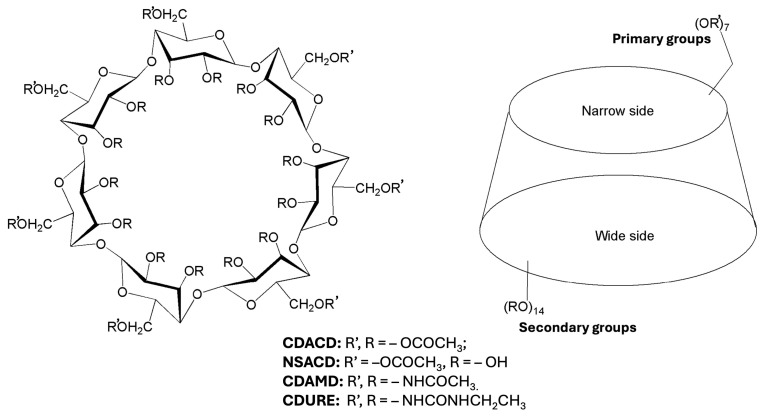
Schematic representation of β-cyclodextrin structures and definition of the systems investigated in this work, based on the type of substituents.

Since the early 2000s, the functionalization of sugars with acetate groups has been one of the design choices for synthesizing molecules that show high solubility in carbon dioxide, in the liquid and supercritical phases [11,12]. In the case of smaller molecules, such as sorbitol and β-D-galactose, reasonably low pressures were sufficient to obtain miscibility, while macromolecular systems, such as peracetylated cyclodextrins (CDACDs), would require access to slightly higher pressure regions of the phase diagram of the fluid [12,13]. Cyclodextrins are macromolecules forming truncated cone structures comprising, for the most common systems, six, seven or eight glucopyranose units corresponding to α-, β- and γ-cyclodextrin, respectively. The native-OH groups of glucose units can be replaced using different substituents, among acetyl groups, as in the peracetylated system. A schematic description of the structure of β-cyclodextrins with different substituents is displayed in Figure 1.

The solubility of peracetylated CD has been observed to be comparable to that of poly(vinil)acetate, the most widely used non-fluorinated polymer in scCO_2_ [13]. The role of the acetyl group in providing enhanced solubility of sugars has been extensively studied. Ranging from the case of poly(vinyl acetate) [14,15,16] to peracetylated cyclodextrins [13,17], the carbonyl group of these units displays a high CO_2_-philic character, based on Lewis acid–Lewis base interactions that may be corroborated by the formation of a weak hydrogen bond between the methyl H atoms and the oxygen atoms of CO_2_.

In the recent two decades, an increasing molecular understanding of the behavior of CDACD has inspired a strategy based on the use of cyclodextrins as solubilizers of nonpolar, hydrophobic species in water [18] to promote the vectorization of molecules, particularly those of pharmaceutical interest, in the scCO_2_ medium [19,20,21,22,23,24].

Additional sustainable applications of scCO_2_ technology are attracting growing interest in the food industry and in biomass conversion [11,25]. Fruit sugars [26,27], fermentable sugars [28,29] and polysaccharides [30], which can be extracted without the use of toxic solvents under relatively mild conditions. scCO_2_ extraction is one of the environmentally friendly methods of choice for producing lignocellulosic biofuels and biochemicals. The conversion of lignocellulosic biomass into glucose monomers remains a costly step in the process. Leveraging the dual role of scCO_2_ as a solvent and a catalytic acid, Kim et al. proposed a method to enhance the hydrolysis of cellulose into glucose [31]. Additionally, scCO_2_ can be employed in the pretreatment of biomass prior to enzymatic hydrolysis to facilitate sugar extraction [32].

Over the recent years, peracetylated sugars have been the primary focus of studies in scCO_2_. However, the influence of different substituents on solubility has also been investigated. Haines et al. reported a decrease in solubility when the α-hydrogens of the acetyl group were substituted with methyl groups (e.g., in the propionyl, butyryl, and isobutyryl groups) [33] Conversely, an increase in solubility was observed for the trimethylacetyl derivative. This observation seems inconsistent with the expected stabilizing effect of a weak H-bond between the methyl hydrogen atoms of the acetyl group and the oxygen atoms of carbon dioxide. However, it supports recent findings, suggesting that enhanced solubility can be achieved with branched compounds [34].

Notably, the successful synthesis of CO_2_-philic compounds featuring two acetylated arms and a urea-based core has been achieved, offering further opportunities for diversification in chemical design within the supercritical medium [35]. Ureido cyclodextrins have also been synthetized using ’one-pot’ approaches based on the Staudinger–aza-Wittig reaction, in which scCO_2_ is simultaneously used as a solvent and a reactant [36]. In a very recent work, this approach was extended by incorporating a mechanochemical procedure, avoiding long reaction times or harsh reaction conditions [37].

Theoretical studies have helped in shedding light on the nature of the interactions between CO_2_ and CO_2_-philic molecules (see ref. [10] for a review). In the case of compounds containing carbonyl groups, ab initio calculations provided, since the 1990s, an interpretation based on weak yet stabilizing electron-donor–electron–acceptor intermolecular interactions [8,38,39,40]. Motivated by some experimental studies, showing the solvating power of scCO_2_ towards amides and substituted urea [41,42,43], some of us recently carried out quantum chemical calculations, demonstrating that the conjugated π system involving the O, C and N atoms represents an additional source of interactions with the CO_2_ molecule [44]. Similarly to what was found for ketones and aldehydes [45], some cooperative interaction was observed and, additionally, a hydrogen-bond involving the acidic N-H proton could be formed. This is consistent to findings based on classical molecular dynamics (MD) simulations of molecules containing these groups [46].

Simulations based on classical force fields are one of the methods of choice to describe the fascinating properties of cyclodextrins [47], particularly to investigate their unique capability to form host–guest complexes [48]. However, MD studies focusing on cyclodextrins in scCO_2_ are still limited. Our past work revealed insights into cavity accessibility, conformational dynamics, and complexation ability of peracetylated systems [23,49]. Antipova et al. have explored the formation of a complex with naproxen in the presence of a cosolvent [50]. In this work, we extended our previous investigations by comparing the behavior in scCO_2_ of CDACD [49] with that of cyclodextrins substituted with amide groups (CDAMD) and urea groups (CDURE) (see Figure 1 for a summary of the acronyms). We focused on β-cyclodextrins, which are the most studied for scCO_2_ applications [17,23,24,36,37,50,51].

To complete our study, we performed MD simulations of peracetylated α- and β-D-glucose (AGLU and BGLU, respectively) in the supercritical medium, the solubility of which has been proven experimentally [11]. To the best of our knowledge, this is the first MD study analyzing the solvation properties of these systems in supercritical carbon dioxide.

This paper is structured as follows. The results are presented and discussed in Section 2. Section 3 outlines the computational protocol employed for the simulations. Section 4 provides the conclusions and highlights potential perspectives arising from this work.

## 2. Results and Discussion

We start the discussion by comparing the results obtained in this work for CDACD, based on longer simulations, with those published in ref. [49]. When monitoring the conformations of the glucopyranose rings in each structure, we observe a conversion from chair to skewboat in some of the units, thus confirming our preceding findings and their consistency with what had been observed in an experimental study [17]. In addition, X-ray measurements on an inclusion complex of permethylated β-cyclodextrin also showed a distorted cavity occurring concurrently with the conformational change of one of the seven identical units to skewboat [52]. On a longer timescale compared to the previous 10 ns simulations, we see that more than one unit can undergo this transition. In particular, Unit 5 is in a skewboat configuration about 40% of the time, with transitions that can last between 500 and 1000 ps (unit numbering is arbitrary, since they are all equivalent). On the other hand, Unit 7 can be found in a skewboat transition about 10% of the simulated time. Some isolated transitions to the boat conformation are also observed. Results for the distribution of dihedrals for each of the seven pyranose units are reported as Supplementary Materials. To complete this discussion, it is important to mention that a very similar conformational dynamics was observed in a very recent MD study of α-cyclodextrin in water [53]. One important conclusion of that work was that, even on a 200 ns timescale, the time window is too limited for a quantitative characterization of the observed transitions. Based on 500 ns simulations, Suarez et al. showed that the convergence of the conformational entropy of α-, β-, γ-cyclodextrin in water strongly depends on the motion that is analyzed, and endocyclic torsions belong to those motions for which conformational entropy oscillates the most [54]. Equilibrium simulations are possibly not suitable to provide a quantitative description of this phenomenon, and biased strategies should be developed ad hoc, which is beyond the scope of this work. However, all reports are consistent with lower conformational dynamics when inclusion complexes are formed [23,53,54].

**Figure 2:**
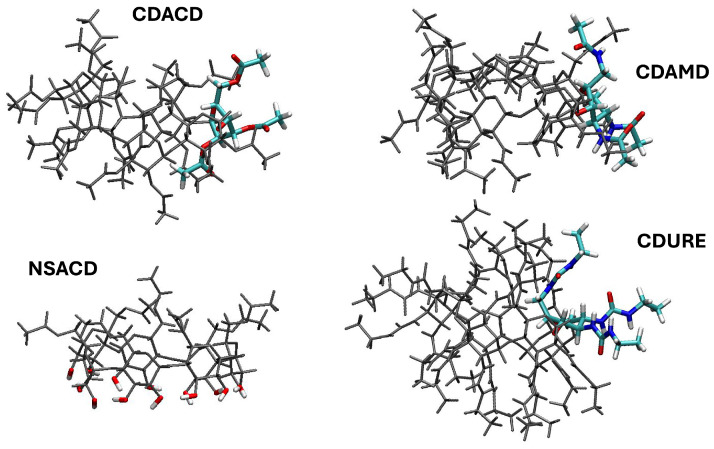
Illustration of the distortion of some pyranose units along the trajectories. We remind that the units are all identical; numbering is thus arbitrary. The overall structure of the macrocycle is displayed in gray, while the chosen units are reported in a licorice representation (C atoms in cyan, O atoms in red, N atoms in blue, H atoms in white). **Top left panel**: Unit 5 of CDACD. **Top right panel**: Unit 4 of CDAMD. **Bottom right panel**: Unit 1 of CDURE. In the **bottom left panel**, we highlight the configuration of the OH groups of NSACD.

We confirmed that one possible path toward a hindered cavity is linked to the chair to skewboat transition. As a matter of fact, this transition is accompanied by a reorientation of one of the acetyl secondary groups of the distorted unit toward the center of the cavity, as illustrated in the top left panel of Figure 2.

**Figure 3:**
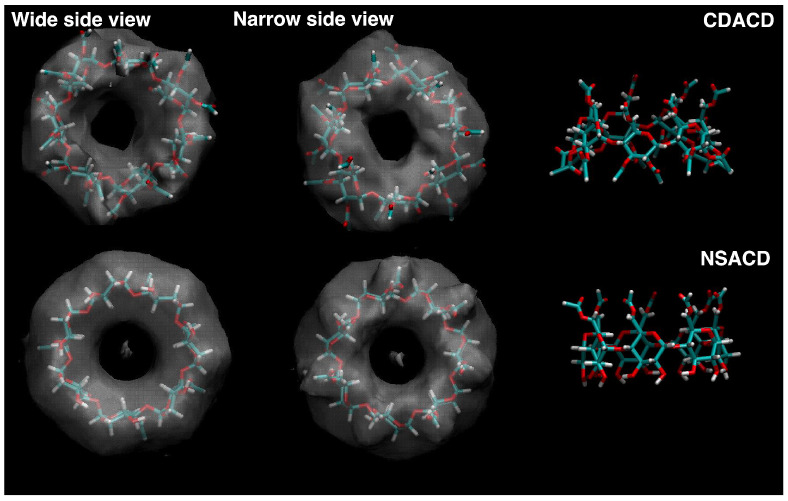
Spatial distribution function (**left**) of the C atom of CO_2_ molecules around the cyclodextrin molecule, represented as a white transparent surface, and average structure of the macrocavity (**right**). **Top figures**: CDACD; **bottom figures**: NSACD. The atom color is the same as defined in Figure 2.

The mobility of the acetyl groups and the distortion of the overall structure of the macrocavity induce partial cavity closure in this system [49,53]. To gain a more complete picture of this phenomenon, we computed the spatial distribution function (SDF) of the C atom of CO_2_ with respect to the CDACD structure. The SDF is reported in the upper left panel of Figure 3, using two different viewpoints and superposed to the average structure of the cavity along the MD trajectory.

This illustration is consistent with a situation in which the solvent molecules can interact with the acetyl groups but cannot access the center of the cavity, at least not in an average picture. The average structure of the molecule (top right panel of Figure 3) suggests a deformed structure compared to that of a native cyclodextrin in water, which is still in agreement with our previous analysis. We shall not investigate this point further, since a detailed analysis was already performed in our previous work.

**Figure 4:**
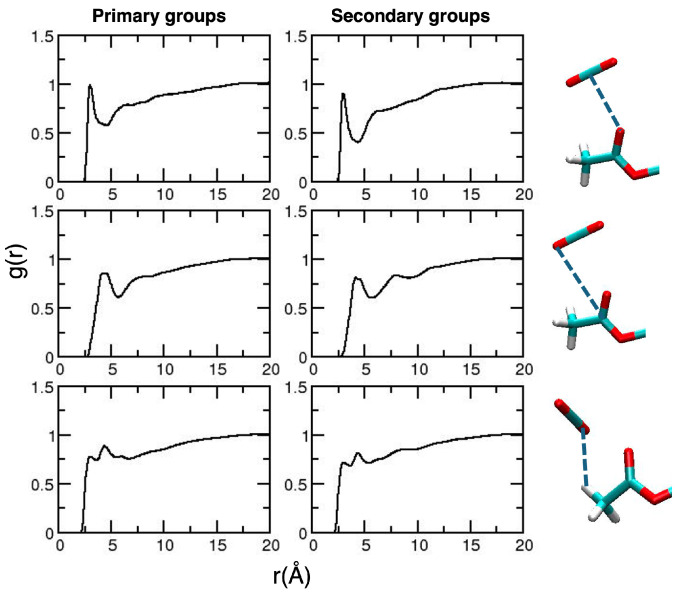
Radial distribution functions for the relevant CDACD–solvent interactions. Results for the acetyl groups of the primary and secondary groups are reported in the **left** and **right panels**, respectively. For each line, the corresponding interactions are depicted in the images on the right-hand side of the Figure (dashed blue lines). The atom color is the same as defined in Figure 2.

After validation of the general picture for the description of CDACD, we move to the analysis of solute–solvent local interactions based on radial distribution functions (RDFs). The collection of relevant results is reported in Figure 4.

The interaction occurring at the closest distance is that between the O atom of the carbonyl groups and the C atom of CO_2_, an interaction that is considered as the signature of the CO_2_-philicity of the system [38,45]. The first peak of the RDF is located at 3.0 Å for the primary groups and at 2.93 Å for the secondary groups. A less structured peak, at larger distances, is observed for the interaction between the C atom of the carbonyl group and the O atom of CO_2_. Interestingly, on a longer timescale, we observed some structuring on the RDFs describing the interactions between the O atoms of CO_2_ and the methyl H atoms. The first peak occurs at 3.0 Å, and this is consistent with quantum chemistry calculations on model systems [38,45], predicting a weak C–H⋯O hydrogen bond [55]. Such an interaction is not observed in the analysis of shorter MD trajectories [49].

To investigate the structural effects of a partial substitution in a native cyclodextrin, we designed a new system in which acetylation is present on the narrow side (whereas the secondary groups are not acetylated) and immersed it in the supercritical medium. Our inspection of the dihedrals revealed no conformational changes (the distribution of the dihedrals is reported as Supplementary Materials). The OH groups form a relatively rigid network of intermolecular hydrogen bonds, keeping the wide side open (see illustration in Figure 2, bottom left panel). This property leads to a macrocavity that stays open, thus allowing a permanent access to the cavity center to solvent molecules. The SDF of C atoms around NSACD clearly displays the presence of such molecules along the whole simulations (bottom left panels in Figure 3).

**Figure 5:**
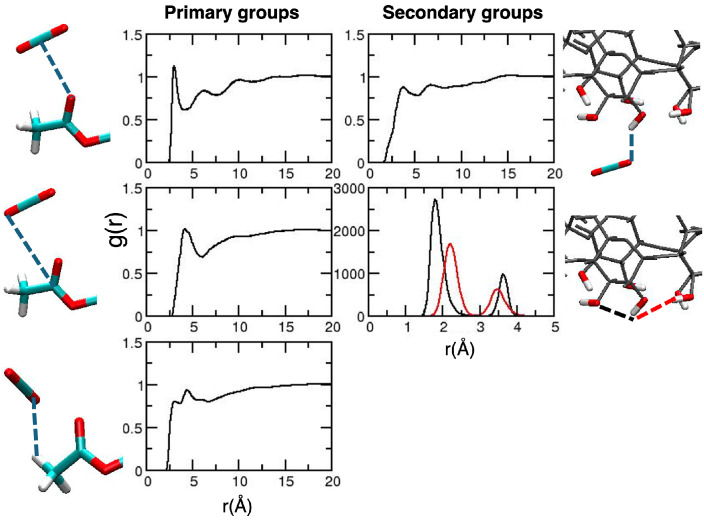
Radial distribution functions for the relevant NSACD–solvent interactions. Results for the primary groups are reported in the **left panels**, along with an illustration of the corresponding interactions (in the images on the left, using dashed blue lines). In the **top right panel**, we report the interaction characterizing secondary groups (see images on the right). Finally, the intramolecular HO⋯HO interaction is reported in the **middle right panel**, using a black line for the interactions within the same pyranose unit and a red line for the interactions between two neighbor units (illustrated as dashed lines of matching color in the image on the right). The atom color is the same as defined in Figure 2.

The RDFs obtained for the primary acetyl groups are very similar to those computed for CDACD (Figure 5, left panel). Some structuring is observed around the H atom of the secondary hydroxyl groups though the first peak occurs at quite large distances (3.7 Å). We also reported an intermolecular RDF for the interaction between the H atom of one hydroxyl group with the O atom of another group. The closest interaction occurs within the same pyranose unit (peak at 1.8 Å); for the two neighbor units, the peak occurs at (2.2 Å).

**Figure 6:**
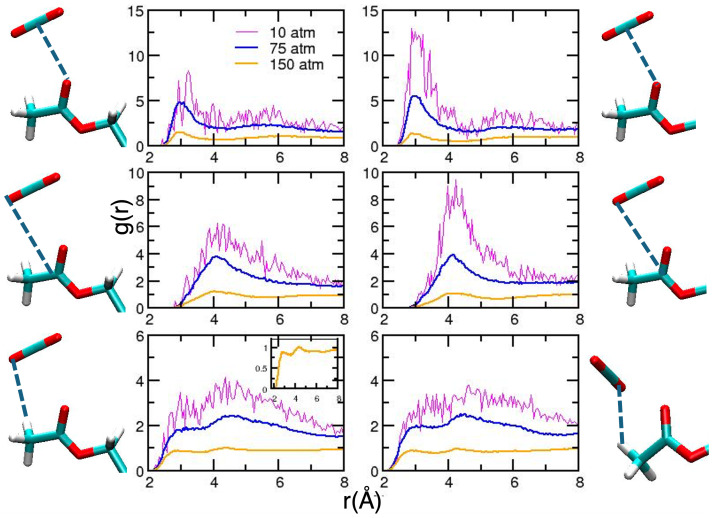
Radial distribution functions for relevant AGLU–CO_2_ interactions. On the left-hand side, we report the curves obtained for the acetyl group attached to the C6 atom [56], whereas those on the right-hand side are averages from the results obtained for the other acetyl groups of the glucose unit. Magenta, blue and orange lines refer to simulations performed at 10, 75 and 150 atm. Atom numbering is reported as Supplementary Material and the atom color is the same as defined in Figure 2.

**Figure 7:**
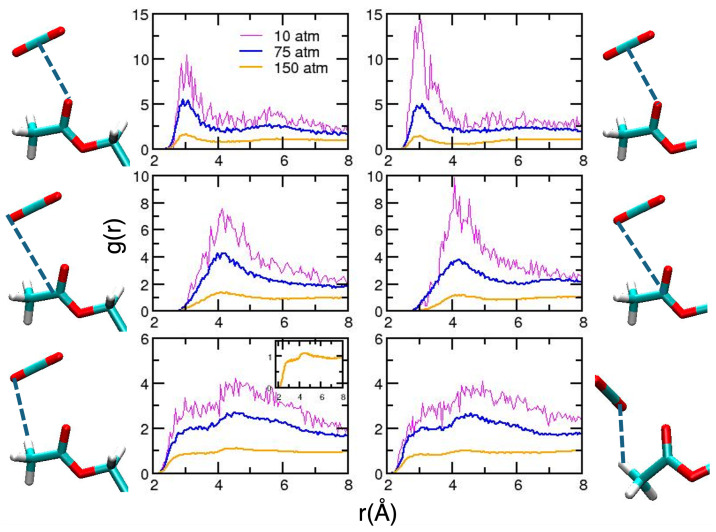
As in Figure 6, for BGLU.

To conclude the discussion involving acetylated systems, for which some experimental data are available [11,13], we report our results for AGLU and BGLU. For all the considered pressures, we did not observe any conformational changes (results are not reported for the sake of simplicity). Therefore, we can safely assume that the changes observed in some of the CDACD units are linked with the complex structural dynamics of the cyclodextrin macrocycle. Radial distribution functions are reported in Figure 6 and Figure 7, displaying the selected intermolecular interactions. We note that the results obtained at the lowest density are very noisy, since the number of molecules per unit volume is very low in these conditions. The overall picture for the acetyl–CO_2_ interaction is similar for the two molecules, for which we observe more structured peaks as density increases. The position of the first peak falls at shorter distances for higher densities.

**Figure 8:**
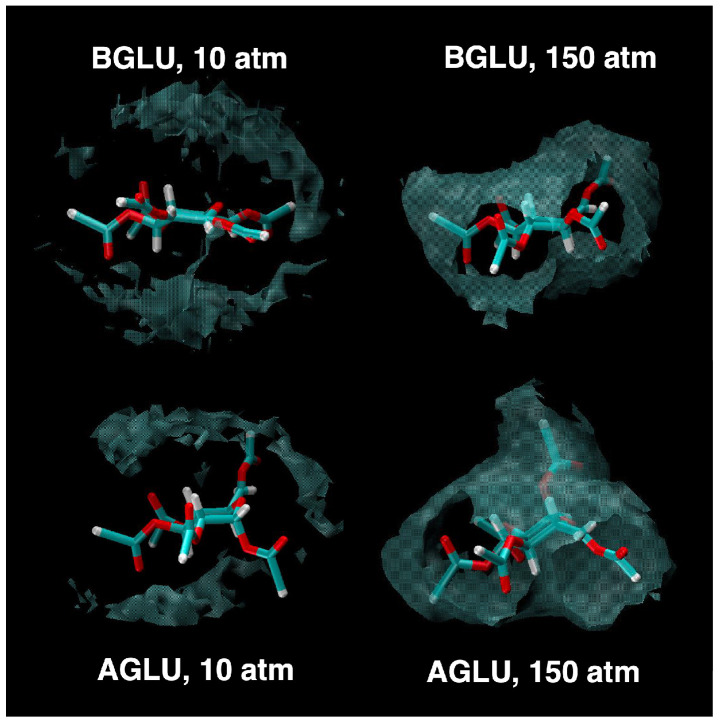
Spatial distribution function of the C atom of CO_2_ molecules around peracetylated α- and β-glucose, reported as a white transparent surface, at different pressures (T = 313 K). The atom color is the same as defined in Figure 2.

However, one subtle difference was observed in the interaction of the H atoms form the methyl groups at a higher density. This is displayed in the inset of the corresponding curves. For AGLU, the RDF is more structured for distances corresponding to first neighbors (first peak at 2.93 Å) compared with BGLU. As a matter of fact, differences in the C–H⋯O network around the two isomers was suggested to be one of the reasons for the different solubility observed in scCO_2_ [11]. The shape of the solvation shell, illustrated using the computed SDF (Figure 8) at two different densities, is also different for the two molecules.

In the CDAMD system, we observed some configurational changes in one of the pyranose units (dihedral distributions reported as supplementary material). However, in this case, the transition to skewboat did not imply the reorientation of secondary substituted groups (see illustration at the top right panel of Figure 2). In addition, they were limited to about 10% of the overall trajectory, and the transitions lasted 200 ps at most.

**Figure 9:**
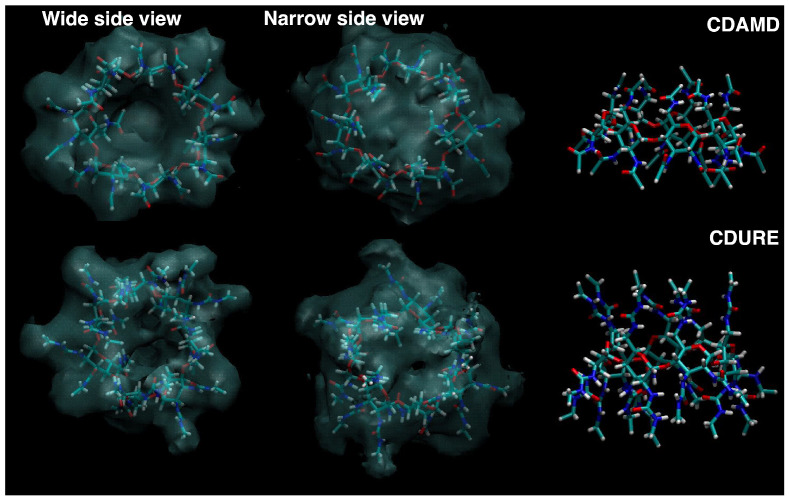
Spatial distribution function (**left**) of the C atom of CO_2_ molecules around the cyclodextrin molecule, reported as a whit transparent surface, and average structure of the macrocavity (**right**). **Top figures**: CDAMD; **bottom figures**: CDURE. The atom color is the same as defined in Figure 2.

The shape of the macromolecule is quite different compared with CDACD. The average structure reported on the top right side of Figure 9 hints at a strong interaction among the primary amido groups forming a cap structure on the top of the cavity.

As a consequence of this tendency, the solvent is present around the amido group, but not in the center of the cavity, and overcrowds the region where the primary amido groups interact, as illustrated in the two top left panels of Figure 9.

**Figure 10:**
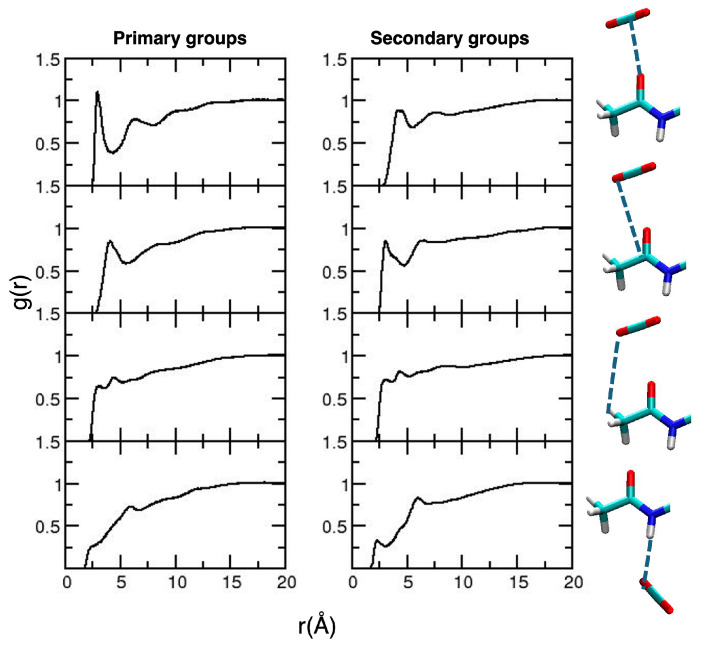
Radial distribution functions for the relevant CDAMD–solvent interactions. In the first three lines, results for the carbonyl groups of the primary and secondary groups are reported in the **left** and **right panels**, respectively. For each line, the corresponding interactions are depicted in the images on the right-hand side of the Figure (using dashed blue lines). On the last line, we report the interactions involving the NH bonds. The atom color is the same as defined in Figure 2.

We analyzed the RDFs for local solute–solvent interactions, reported in Figure 10.

The results involving the carbonyl group interacting with CO_2_ molecules are very similar to those obtained for CDACD. In addition, it is important to note that the interactions involving the acidic HN atoms of the primary amido groups lead to less structuring compared to secondary groups. The former groups are longer and more mobile; amido groups are mobile and participate to a much larger set of interactions. In particular, given their position with respect to the glucopyranose rings, the N–H bonds are less exposed to the solvent than the carbonyl and methyl groups, occupying more periferical positions. To confirm this interpretation, we computed the HN⋯N RDFs for atoms belonging to different pyranose groups, which exhibit broad peaks between 3 and 4 Å (reported in the Supplementary Materials Section).

In the CDURE simulations, conformational changes are observed in two units (see the Supplementary Materials Section for the dihedral distributions). We noted that in one of them, the most important conversion was chair to boat, and that this situation was related with a partial tumbling of the distorted pyranose unit around the C–O bonds linking it to the two neighbor units (see illustration on the bottom right panel of Figure 2). For this unit, the transitions occur on about 6% of the full trajectory, lasting about 500 ps. The average cavity shape is quite deformed (bottom right panel of Figure 9), and the solvent is mostly outside of the cavity, surrounding the ureido groups (same Figure, bottom left panels).

**Figure 11:**
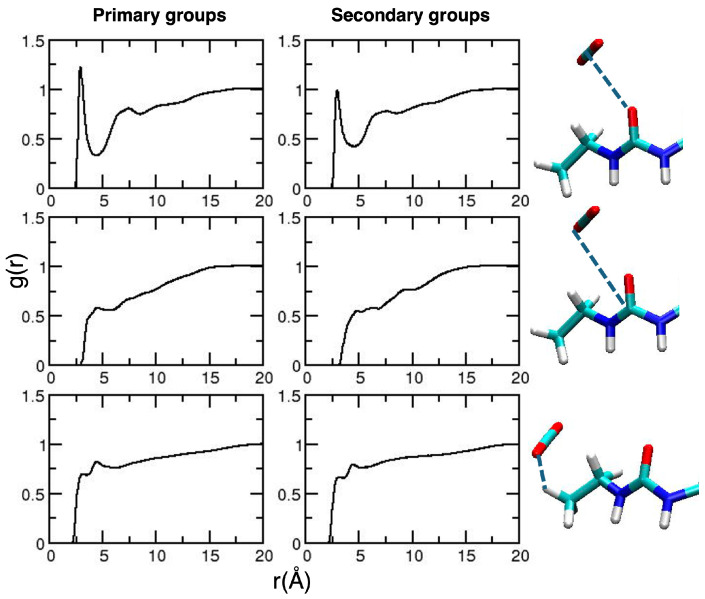
Radial distribution functions for the relevant CDURE–solvent interactions. In the first three lines, results for the carbonyl groups of the primary and secondary groups are reported in the **left** and **right panels**, respectively. On the last line, we report the interactions involving the methyl groups. For each line, the corresponding interactions are depicted in the images on the right-hand side of the Figure, using dashed blue lines. The atom color is the same as defined in Figure 2.

The analysis of the radial distribution functions applied to the acetyl–solvent interactions is quite similar to that presented for CDAMD (Figure 11).

**Figure 12:**
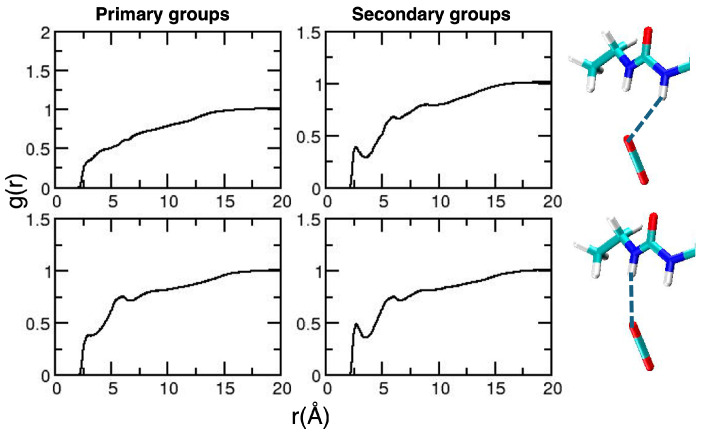
Radial distribution functions for the relevant CDURE–solvent interactions involving the NH bonds of the ureido groups of the primary groups (**left panels**) and of the secondary groups (**right panels**). For each line, the corresponding interactions are depicted in the images on the right-hand side of the Figure. The atom color is the same as defined in Figure 2.

For the ureido substituent, two NH sites are available. Therefore, we decided to analyze the interactions involving each of them separately, reported in Figure 12.

Structuring is observed around the NH groups of the secondary units, which are more exposed to the solvent, being less involved in intramolecular interactions with the groups of neighboring units, as shown by an inspection of the intramolecular RDFS (see the Supplementary Materials Section).

In concluding this section, we compare the average solute–solvent interaction energies computed for CDACD, CDAMD, and CDURE, as presented in Table 1. It is important to note that these are approximated results, since Coulombic and Lennard–Jones interactions were cut off at a half of the simulation box in each direction, thus neglecting long-range effects. Although more accurate analyses, such as those based on solvation free energies, could provide better predictive value, methods like thermodynamic integration are computationally intensive, require specific thermodynamic cycle definitions, and pose challenges in complex systems [57], as in the case of supercritical fluids. Consequently, since our focus is on solvation structures, such analyses are beyond the scope of this work.

Our findings indicate more favorable interactions for the amido and ureido systems compared to the acetylated one, for which solubility data are available. Additionally, the interaction energies calculated for AGLU and BGLU at 150 atm align with expectations, reflecting a more favorable AGLU-scCO_2_ interaction, as expected considering the structure of the solvation shell analyzed above.

## 3. Materials and Methods

Classical MD simulations were performed using Amber 2016 [58] and visualized by means of VMD [59] In our previous studies on cyclodextrins [23,49], we adopted the approach developed by Cézard et al. [47]. This method involves the creation of a unified force field, termed “q4md-CD”, which integrates elements from both the GLYCAM04 [60,61,62] and Amber99SB [63] FFs. This approach takes advantage the structural similarities among various cyclodextrins, ensuring reliable transferability of parameters [48]. Our prior research on CDACD in supercritical carbon dioxide validated this strategy, demonstrating good agreement between simulated and experimental data describing the structural and complexation characteristics of the macrocycle. The EMP2 force field was used for CO_2_, ref. [64] which was chosen because of its ability to reproduce the thermodynamic properties of CO_2_ in the near critical phase.

We carried out simulations of peracetylated β-cyclodextrin, and the corresponding systems using an amido and an ureido group replacing the acetyl group, in supercritical CO_2_. An additional macrocavity, presenting acetylation on the narrow side and native-OH groups on the wide side was considered as well. Simulations of α-glucose and β-glucose in scCO_2_ were also carried out (a scheme of the molecular structures is reported as Supplementary Materials). Details about the systems definition and acronyms are provided in Figure 1.

Simulations were run in cubic boxes, and periodic boundary conditions were taken into account with the particle mesh Ewald method [65] for treating long-range electrostatic interactions, whereas Lennard–Jones intermolecular interactions were cut off at a half of the simulation box. For all cyclodextrins, one macrocavity was inserted in the simulation box and solvated with 4356 molecules, whereas 500 molecules were used for the boxes containing AGLU and BGLU.

After minimization using the steepest descent algorithm, we performed a 2 ns equilibration in the NPT ensemble using Berendsen’s thermostat and barostat [66] with a 1 fs time step. Consistently with our previous work, we equilibrated the cyclodextrin boxes to a pressure of 40 MPa and a temperature of 313 K to achieve similar conditions as those used in experiments for CDACD [13]. To demonstrate that the systems were properly equilibrated, we included an illustration of our analysis in the Supplementary Materials Section. In the case of AGLU and BGLU, we performed our analysis at 313 K and three different pressures (1.0, 7.6, 15.2 MPa, corresponding to 10, 75 and 150 atm), to move along an isotherm of the medium, from the gas phase to the supercritical phase. At the highest pressures, both compounds were shown to be soluble in scCO_2_ [11].

After equilibration, data analysis was conducted by employing the post-processing tools built in the Amber 2016 package, using 50 ns trajectories.

## 4. Conclusions

In this work, we have expanded our previous study [49] on peracetylated β-cyclodextrin by employing larger sampling, which enabled us to reinforce our key findings and achieve a more detailed understanding of specific intermolecular interactions. Beyond the well-established O⋯C acetyl–CO_2_ interaction, we also identified the signature of a weak _2_HCH⋯O methyl–CO_2_ cooperative interaction, consistent with quantum chemistry calculations on model systems [38,45]. Additionally, conformational changes in some units were observed, aligning with NMR measurements [17].

We then investigated two additional cyclodextrins, substituted with amido and ureido groups, which have been suggested to exhibit CO_2_-philic character [10]. Conformational changes were also observed in these derivatives, and the fundamental solute–solvent local interactions identified in CDACD were similarly present.

In all three cases, solvent molecules were found to associate with the CO_2_-philic groups but did not stably occupy the center of the macromolecular cavity. Solvent accessibility appears to be more influenced by the dynamics and intramolecular interactions of bulky substituents than by conformational changes within the glucose rings. In a cyclodextrin designed ad hoc with acetyl substituents restricted to the narrow side, the overall dynamics remained relatively undistorted, providing access to solvents to enter the cavity center. The trend in the computed solute–solvent interaction energies suggests that CDAMD and CDURE may exhibit more favorable interactions with supercritical carbon dioxide compared to CDACD.

Finally, we identified distinct differences in the solvation structure of α- and β-glucose that are consistent with their differing solubility behaviors in scCO_2_.

Given the growing interest in the molecular details of sugar–CO_2_ interactions in the supercritical phase—particularly for optimizing extraction technologies [11,25,26,27,28,29,30,31,32]—and the promising results in the sustainable synthesis of substituted cyclodextrins in this medium [36,37], we believe this work provides valuable insights into the solute–solvent interactions that will help drive further advancements in the field.

## Figures and Tables

**Table 1:** Solute–solvent interaction energies computed for CDACD, CDAMD, CDURE, AGLU, and BGLU. In the latter two cases, we reported the results obtained at the highest density. Averages along the simulated trajectories are reported along with standard deviations.

	Interaction Energy (kcal/mol)
CDACD	−174.63 ± 28
CDAMD	−191.91 ± 40
CDURE	−264.63 ± 59
AGLU (150 atm)	−50.00 ± 16
BGLU (150 atm)	−40.47 ± 13

## Data Availability

The original contributions presented in this study are included in the article/Supplementary Materials. Further inquiries can be directed to the corresponding author.

## Supplementary Materials

The following supporting information can be downloaded at: https://www.mdpi.com/article/10.3390/molecules30061256/s1, a scheme of the molecular structures of AGLU and BGLU; the atom numbering for the glucopyranose units; an illustration of the convergency tests; the distributions of dihedrals characterizing the pyranose ring conformations; the intramolecular radial distribution functions for the interactions among NH groups in CDAMD and in CDURE.
