# Supplementary material for: A Molecular Dynamics Study of the Solvation Properties of Sugars in Supercritical Carbon Dioxide
Source: Molecules. 2025 Mar 11;30(6):1256. doi: 10.3390/molecules30061256 (PMC11944854; doi:10.3390/molecules30061256)
Supplement: Supplementary file 1 [file molecules-30-01256-s001.zip › molecules-3481407-supplementary.pdf]

Supporting Information for: A Molecular Dynamics Study of the Solvation Properties of Sugars in Supercritical Carbon Dioxide

Alexandrine Lambert and Francesca Ingrosso *

Laboratoire de Physique et Chimie Théoriques UMR 7019, Université de Lorraine and CNRS,
F-54000 Nancy, France; alexandrine.lambert@univ-lorraine.fr
* Correspondence: francesca.ingrosso@univ-lorraine.fr

We report in the following pages a scheme of the molecular structures of AGLU and BGLU; the atom numbering for the glucopyranose units; an illustration of the convergency tests; the distributions of dihedrals characterizing the pyranose ring conformations; the intramolecular radial distribution functions for the interactions among —NH groups in CDAMD and in CDURE.

Labels

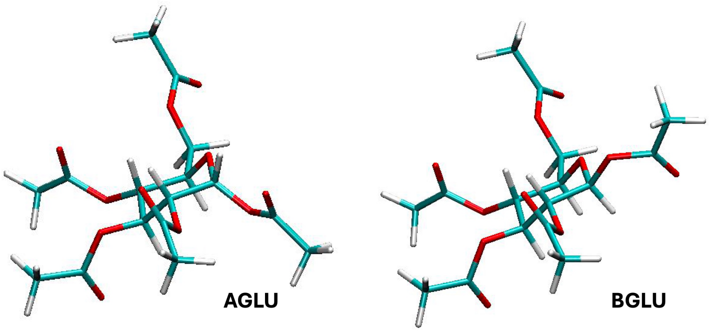

Figure S1: Structure of peracetylated α— and β—glucose (AGLU and BGLU, respectively).

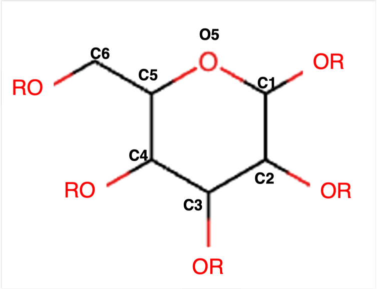

Figure S2: Definition of the atom numbering used for the glucopyranose unit.[1]

Convergency tests

We report the most relevant results obtained on the NPT equilibration and on a block analysis on the production run, conducted on CDURE in supercritical CO_2. The results obtained for the other units are very similar and will not be reported for the sake of simplicity.

In Figure S3 we report an image illustrating the simulation box.

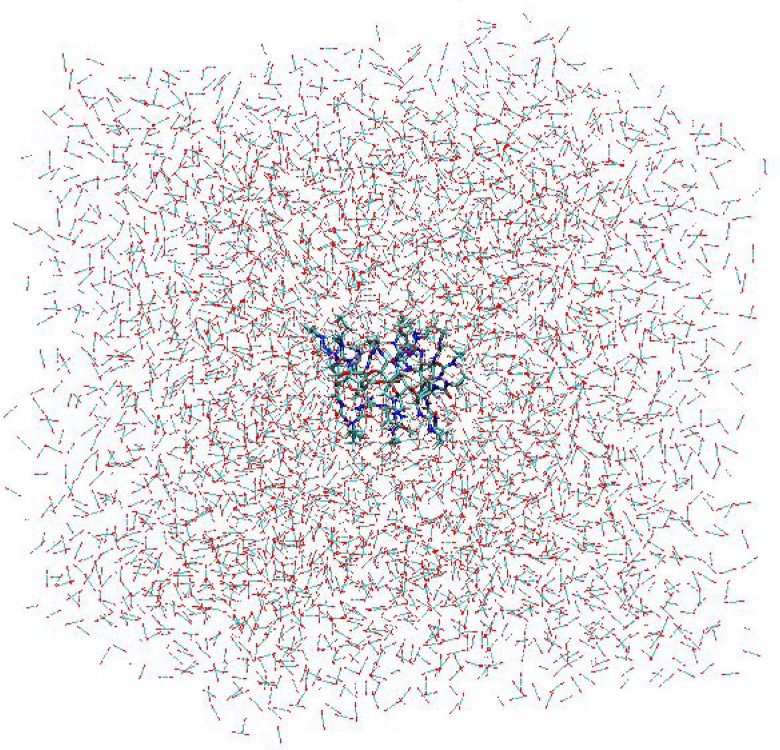

Figure S3: CDURE: snapshot illustrating the cubic simulation box.

In Figure S4 we report the evolution of relevant thermodynamic descriptors in the 1 ns NPT trajectory run in the equilibration. We note that the initial value of the box size was taken from our previous work on CDACD, providing a reasonable starting point to achieve the target 40 MPa and 313 K conditions, when using the same number of solvent molecules. The final value of the box volume, to be used in the NVT equilibration and in the production run, was computed as the average volume along the last 300 ps in the NPT run.

In Figure S5 we the results obtained for the radial distribution function describing the

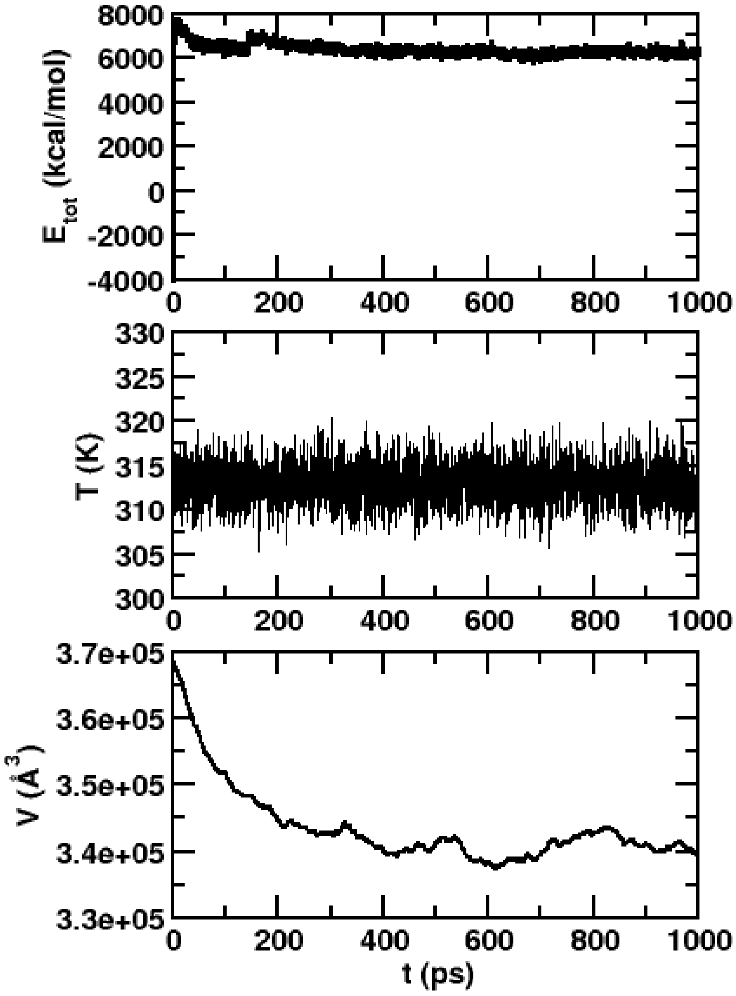

Figure S4: CDURE: Evolution of the total energy (top), the temperature (middle) and the box volume (bottom) along the 1 ns NPT equilibration.

main CDURE-CO_2 interaction, computed along 2 ns blocks, into which the overall 10 ns trajectory was divided. The average value over the five blocks obtained for the first peak maximum is 1.225 Å and the standard deviation is 0.02 Å.

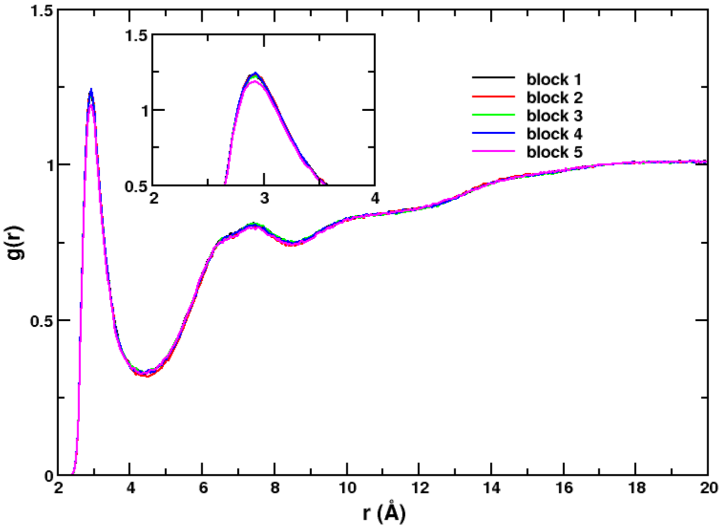

Figure S5: CDURE: Block analysis applied to the radial distribution function describing the local interactions between the O atom of the carbonyl groups on the urea substituents and the C atom of carbon dioxide. The inset shows a magnification of the top of the first peak.

In Table S1, we report the analysis of the total interaction energy computed between CDURE and the solvent in terms of each 2 ns block and of the total 10 ns trajectory.

Table S1: CDURE: solute-solvent interaction energies. Results reported for the five 2 ns blocks and for the total 10 ns trajectory are reported.

	Average (kcal/mol)	St. dev. (kcal/mol)
Block 1	-267.99	± 64
Block 2	-261.17	± 59
Block 3	-250.76	± 59
Block 4	-261.76	± 59
Block 5	-280.02	± 52
Block average	-264.34	± 4
Production run	-264.63	± 59

Dihedral distributions

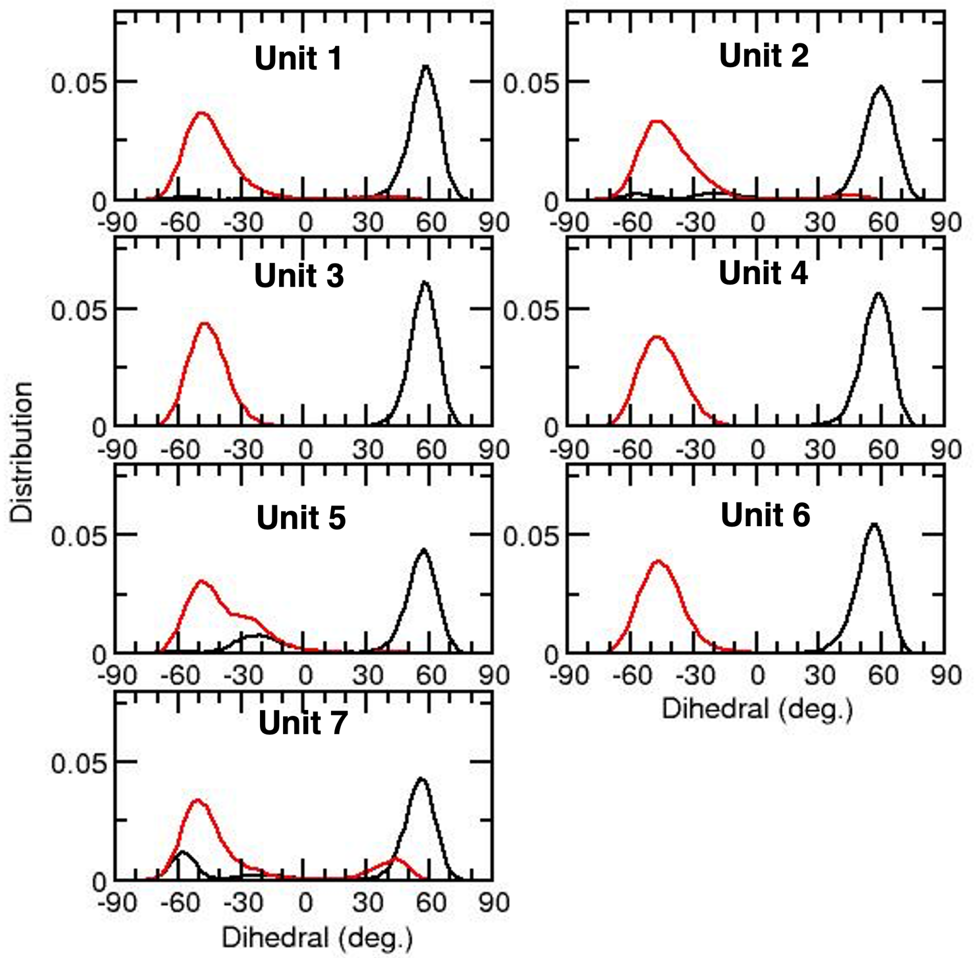

Figure S6: CDACD: normalized distributions of relavant dihedrals. O5—C5—C4—C3 (black curves) and O5—C1—C2—C3 (red curves). The atom numbering is reported in Figure S2. The different plots correspond to the dihedrals of each of the seven pyranose units forming the cyclodextrin macrocavity.

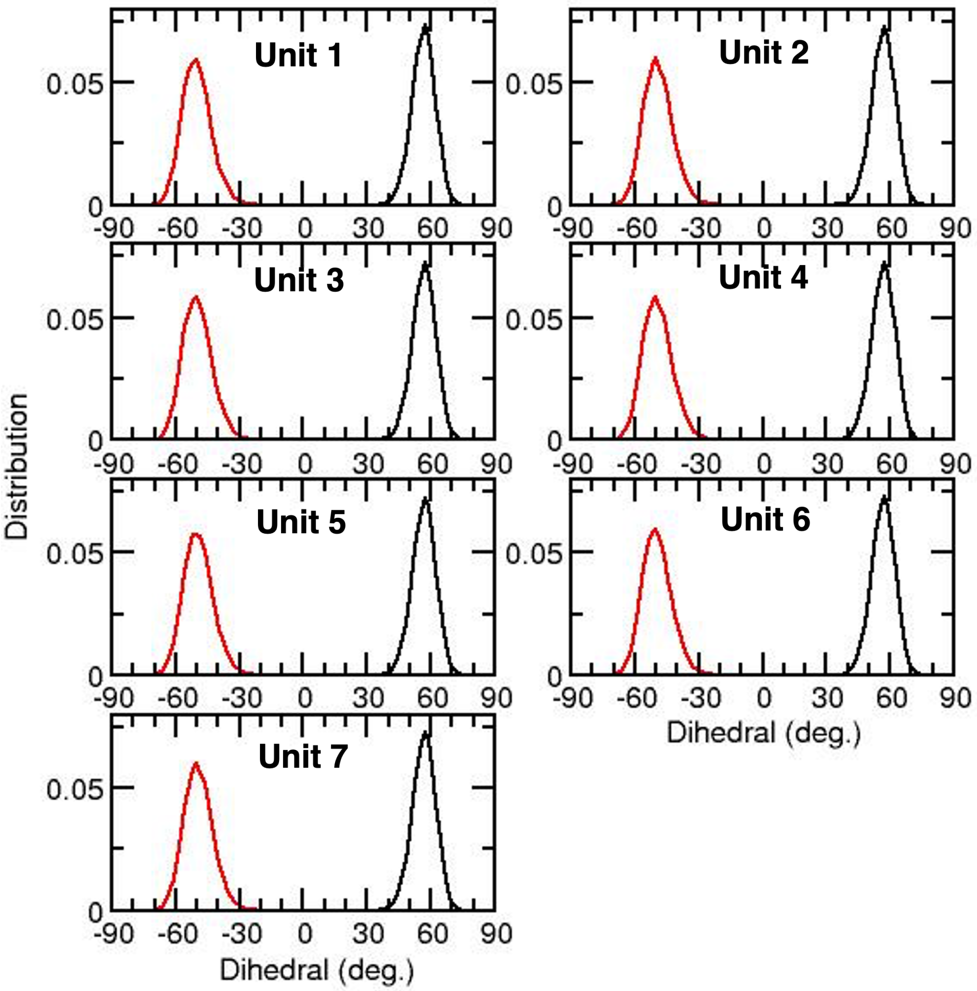

Figure S7: NSACD: normalized distributions of relavant dihedrals. O5—C5—C4—C3 (black curves) and O5—C1—C2—C3 (red curves). The atom numbering is reported in Figure S2. The different plots correspond to the dihedrals of each of the seven pyranose units forming the cyclodextrin macrocavity.

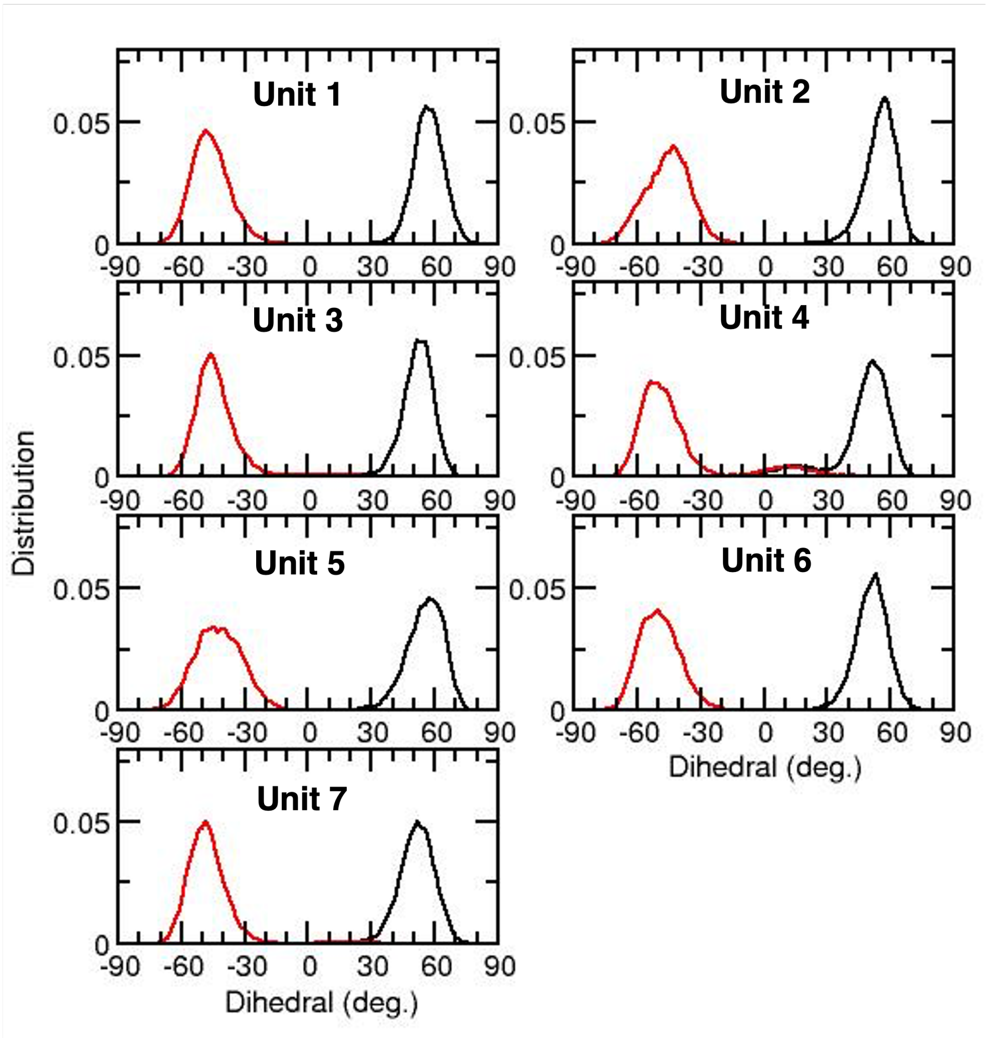

Figure S8: CDAMD: normalized distributions of relavant dihedrals. O5—C5—C4—C3 (black curves) and O5—C1—C2—C3 (red curves). The atom numbering is reported in Figure S2. The different plots correspond to the dihedrals of each of the seven pyranose units forming the cyclodextrin macrocavity.

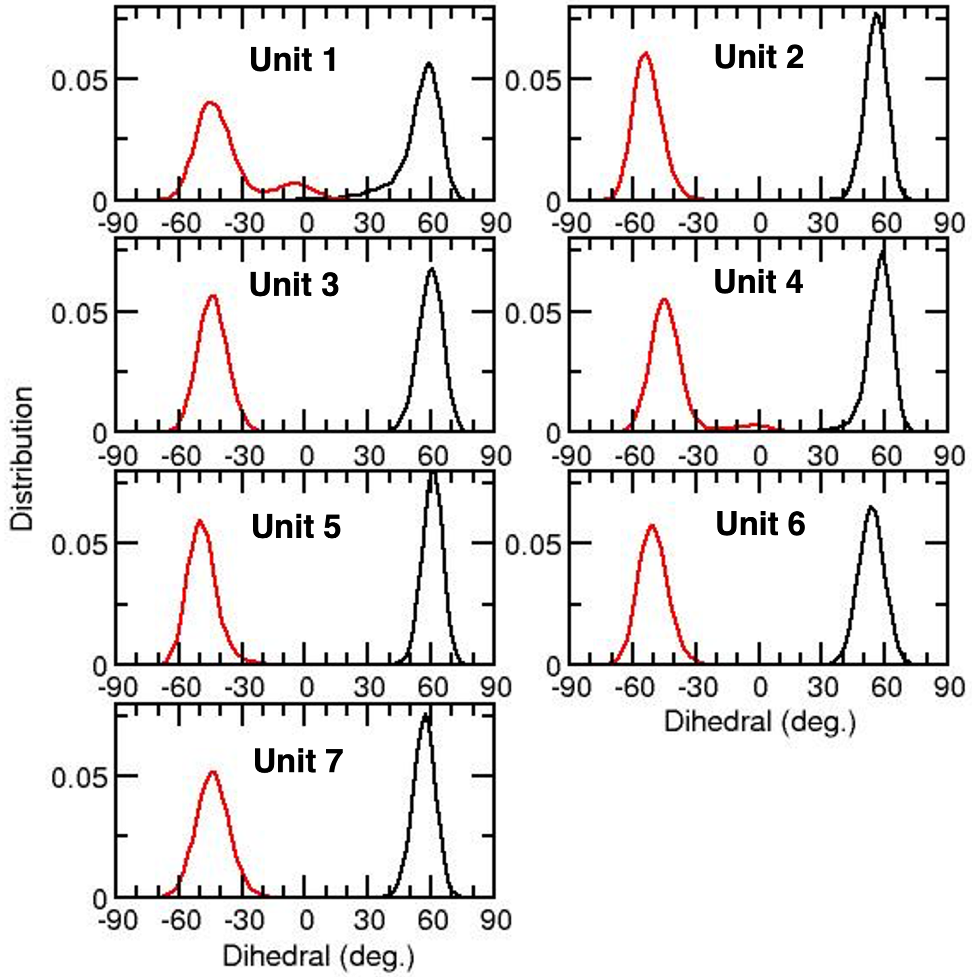

Figure S9: CDURE: normalized distributions of relavant dihedrals. O5—C5—C4—C3 (black curves) and O5—C1—C2—C3 (red curves). The atom numbering is reported in Figure S2. The different plots correspond to the dihedrals of each of the seven pyranose units forming the cyclodextrin macrocavity.

Intramolecular radial distribution functions

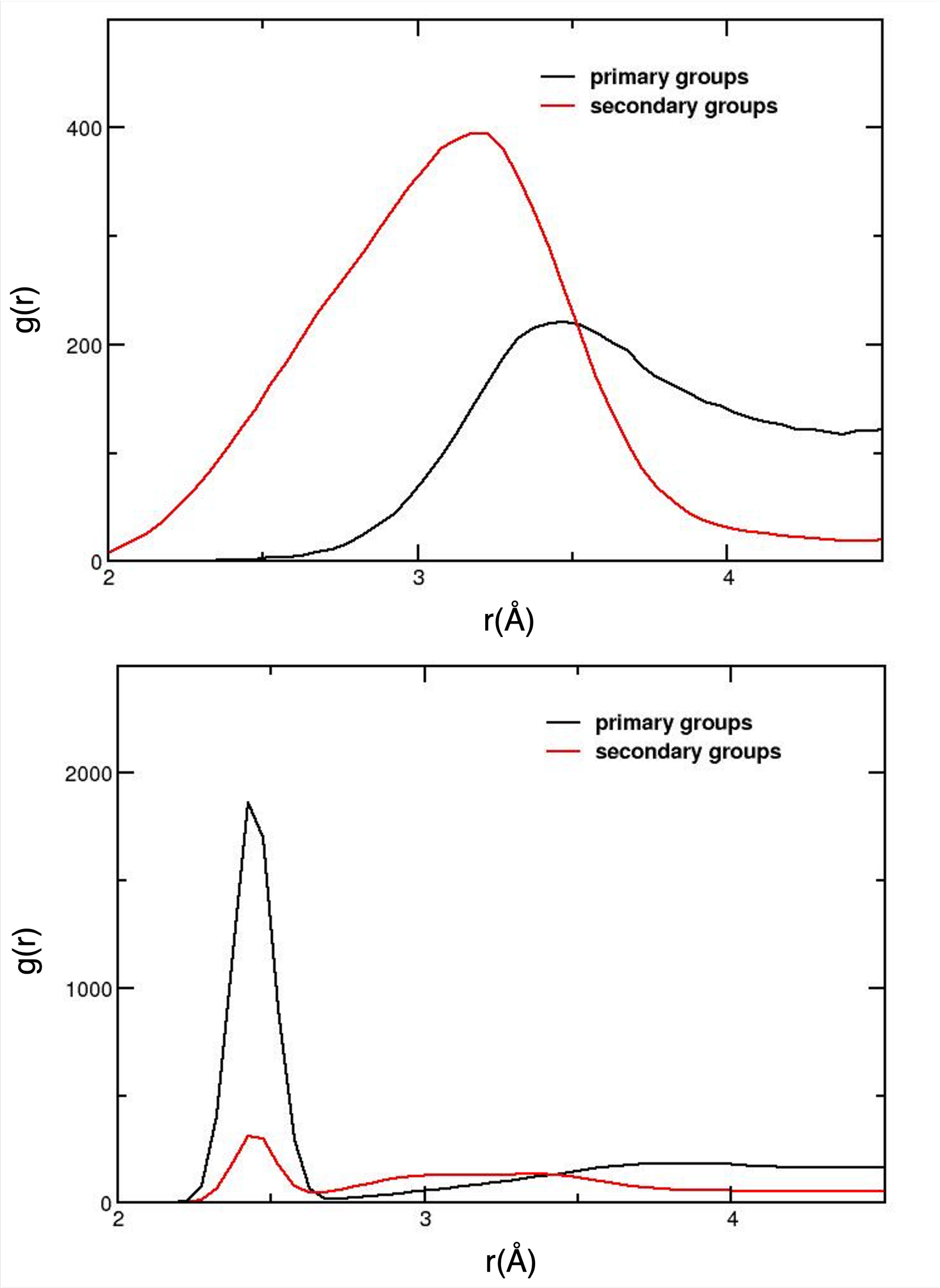

Figure S10: Intramolecular radial distribution functions for the interactions between the HN atoms and the NH atoms of different pyranose units in CDAMD (top panel) and in CDURE (bottom panel).

References

(1) Varki, A. et al. Symbol Nomenclature for Graphical Representations of Glycans. *Glycobiol.* **2015**, *25*, 1323–1324.
